# Correlation between CT - derived bone density and optimal bone densities acquired from CBCT scans

**DOI:** 10.6026/97320630019495

**Published:** 2023-04-30

**Authors:** Saumya Mehta, Subhashree Rohinikumar, Abhinav Rajendra Prabhu, Thiyaneswaran Nesappan, Vishnu Priya Veeraraghavan, Rajalakshmanan Eswaramoorthy

**Affiliations:** 1 Department of Implantology, Saveetha Dental College and Hospitals, Saveetha Institute of Medical and Technical Sciences (SIMATS), Saveetha University, Chennai 600077, India; 2 Department of Biochemistry, Saveetha Dental College and Hospitals, Saveetha Institute of Medical and Technical Sciences (SIMATS), Saveetha University, Chennai 600077, India; 3 Department of Biomaterials, Centre of Molecular Medicine and Diagnostics (COMManD), Saveetha Dental College and Hospitals, Saveetha Institute of Medical and Medical and Technical Sciences, Saveetha University, Chennai 600077, India

**Keywords:** Hounsfield unit (HU), primary stability, dental implants, osseo-integration

## Abstract

It is of interest to explore the correlation between preoperative bone density, assessed via CBCTT, and primary stability of dental implants, assessed by torque ratchet. A total of 100 patients who had implant placed were taken a sample for this retrospective study. The Hounsfield units (HU) derived preoperative bone densities at implant sites that were acquired with the help of the CBCT and the primary stability was achieved during the day of surgery. Both were compared to optimum bone densities. Statistical correlation was done between the HU and Bone density. Data suggests that evaluating HU values, along with other parameters, before performing implant surgery could lead to better primary implant stability.

## Background:

Dental implants placement has become an important part of clinical practice for rehabilitation of partially and totally edentulous patients because of its benefits [1, 2, 3, 4]. Success of dental implants depends upon osseo-integration and stability at both primary and secondary levels. Primary stability of dental implants depends upon various factors such as Implant length, implant diameter, implant surface treatment and many other macro-geometrical and micro-geometrical characteristics [1, 4]. A pre-operative evaluation of bone quality using subjective radiological rating scales has been suggested since research links low bone quality to a higher likelihood of implant failure [1, 4] The majority of grading scales are based on the description of cortical bone thickness and cross-sectional trabecular morphology. However, there isn't a single, widely acknowledged system for grading the mandibular and maxillary bone quality [1, 4]. Lekholm and Zarb's approach is the most often used for pre-operative implant assessment; it divides bone quality into four groups based on the degree of cortication and trabecular bone morphology [5, 6, 7]. There is a correlation between stability of the implants and bone quality which suggests that surgeons can predict primary stability of implant before its placement and could involve some changes and modifications in the treatment plan such as implant position, healing period, implant selection either during the execution of procedure or prior to it[5, 7]. CBCT technology has advanced rapidly in terms of scientific and technological advancement with a substantial impact on implant dentistry [5, 7]. In this situation, Parsa *et al*. tested CBCT with multislice CT (MSCT) and micro-computed tomography (micro-CT). Their findings showed a strong correlation between MSCT and CBCT, proving that CBCT may be employed at the implant site for evaluating bone mineral density. CBCT has replaced multislice computed tomography (MSCT) despite not having the same accuracy as traditional CT for assessing bone density. These advantages include improved accuracy, less radiation exposure, higher resolution, a shorter procedure time, and lower costs [8, 9, 10, 11].The Hounsfield unit used in CT scans is proportional to the amount of x-ray attenuation that is allocated to each pixel in order to display the image that reflects tissue density. Although CBCT manufacturers and software providers portray grey scales as the HU, the grey scale (voxel value) in CBCT indicates the degree of x-ray attenuation [12, 13, 14].

Misch initially classified bone mineral density as CT values (Hounsfield units) into five ranges to assess bone density, with D1 being the highest with a value >1,250 HU; followed by D2: 850 to 1,250 HU; D3: 350 to 850 HU; and D4: 150 to 350 HU. Additionally, the D1 bone quality was shown to be predominant in the anterior jaw, followed by the D2 and D3 bone qualities in the anterior maxilla and posterior mandible. The posterior maxilla is where D4 bone quality is most frequently seen. It has been acknowledged that primary implant stability is a crucial requirement for achieving osseo-integration later on. Preoperative examination of cortical thickness and trabecular bone HU appears to be the most accurate way for predicting implant stability, according to [15,16,17,18], who demonstrated a substantial linear association between damping values and HU values at implant insertion. They stated that compared to stationary implants, the 3-year survival rate for mobile implants was considerably lower (P<0.001). A more predictable implant treatment procedure may be represented by the capacity to forecast primary implant stability and bone quality during the pre-surgical assessment of the implant placement site [19, 20, 21]. Therefore, it is of interest to assess the preoperative bone densities at implant sites, measured in Hounsfield units (HU), where primary stability has been achieved, and to compare these values with the ideal bone densities.

##  Materials and Methods:

## Patient's selection

The Saveetha Dental College ethics committee received and accepted this study project. The participants were individuals seeking dental implant therapy at the Saveetha dental university. At the time of implantation, the patients included in the study had been missing some of their teeth for at least six months. Patients who demonstrated inadequate motivation or willingness to follow the study's protocol, exhibited signs of alcohol or cigarette abuse, or suffered from bone metabolism disorders were excluded from the study.. A total of 100 implants (n=100) were taken into consideration for the study which were placed into individuals from 15th Jan 2022 to 13 Nov 2022.

Inclusion and Exclusion criteria are as follow:-

## Inclusion Criteria:

[1] Giving consent after being informed

 [2] Partially edentulous arch.

[3] Need of single or multiple implants in the mandible or maxilla

[4] A minimum 6-month history of edentulism in the area to receive implants

[5] Normal Complete Blood Count.

## Exclusion criteria:

[1] Inability or unwillingness to follow the study procedures

[2] Presence of untreated or uncontrolled dental caries or periodontal disease

[3] Known or suspected active cancer

[4] History of chemotherapy within the past 5 years

[5] History of head and neck radiation therapy

[6] History of metabolic bone diseases

[7] Medical conditions that make implant insertion unfavorable

[8] Requirement for systemic corticosteroids

[9] Current or past use of intravenous bisphosphonates

[10] Current or past use of oral bisphosphonates for more than 3 years

[11] History of bone grafting or sinus lift in the planned implant site

[12] Current need for bone grafting or sinus lift in the planned implant site

[13] Ongoing alcohol or drug abuse.

## Bone density:

Using a CT scanner with the following technical specifications: 135 kV, 150 mA, 0.5 s, 0.5 mm slice thickness, 0.3 mm slice increment, and 0°gantry angulation, the bone density of each patient was evaluated prior to implant planning. CT scan data was stored in DICOM format and transferred to DTX planning software where Hounsfield units were measured at the edentulous site at the time of treatment planning. These bone densities were then classified and categorised based on Misch Classification of Bone Density (Table 1).

## Radiological evaluation:

Radiological evaluation was performed by using Orthophos XG3 CBCT machine from Dentsply Sirona and Kodak (CareStream)-9500, 3D Dental imaging software, version 6.14, the patient's maxilla or mandible were subjected to a CBCT scan based on the arch of interest (Saveetha Dental College Radiology lab,chennai). The following were the acquisition criteria: Voxel sizes: 0.5, 0.3, 0.25, and 0.2 mm, 90 kv voltage, 10 Ma current, 10.8 s exposure time, 360-degree rotation, 32-bit depth, and slice thickness of 180 microns. The workstation monitor's generated images were transferred as DICOM files using a CD to a H.P Laptop [Processor 12th Gen Intel(R) Core(TM) i7-12700H 2.30 GHz] RAM 16GB System type 64-bit operating system, x64-based processor Windows 11 Home].

## Implant placement and insertion torque measurements:

A single operator placed all of the implants according to a conventional protocol for implant placement. The operator was not aware of the bone density measurements at the implant sites as they were blinded to the bone analysis. Following implant placement, the stability of each implant was evaluated using a torque ratchet specific to the implant system, and the outcomes were recorded in (Table 2).

## Statistical analysis:

For the statistical analysis, IBM SPSS version 24.0 (SPSS Inc., Chicago, IL, USA) was utilised. Means, standard deviations, standard errors, as well as the median, minimum, and maximum values were computed in a descriptive analysis. One way ANOVA test was performed to analyse the difference in HU and primary implant stability keeping the level of significance at 0.05.

## Results:

## Hounsfield unit obtained from CBCT evaluation:

The Hounsfield unit (HU) values varied depending on the implant placement site, with the highest value observed in the mandibular anterior region at 820 ±150.4. The mandibular posterior region had the second-highest HU value (750.56 ± 135), followed by the maxillary anterior region (691.6 ±154.8) and maxillary molars (594 ± 160.2) as shown in (Table 2).This shows that density of bone is observed to be highest in anterior mandible and least in posterior maxilla.

## Primary implant stability obtained during implant insertion:

Insertion torque was found to be most amongst the anterior mandible (45 +/- 7 Ncm) as compared to other regions. Area which showed the least amount of insertion torque was the Maxillary posterior region which was 32 +/- 3 Ncm.

## Comparison between Hounsfield unit and insertion torque:

For association between Hounsfield unit and primary stability for lower arch, out of 50 implant sites 41.2% of the bony sites having Hounsfield unit in range of 850-1250 HU and 58.8% of the bony sites having more than 1250 HU achieved primary stability between 30 - 40 Ncm. Total of 33 implants achieved more than 45 Ncm of primary stability and 17 implants achieved primary stability between 30-40 Ncm. For association between Hounsfield unit and primary stability for upper arch, out of 50 implant sites 0% of the bony sites having Hounsfield unit in range of 850-1250 HU and 100% of the bony sites having more than 1250 HU achieved primary stability of more than 45 Ncm. Total of 20 implants achieved more than 45 Ncm of primary stability and 30 implants achieved primary stability between 30-40 Ncm.

## Discussion:

The failure rates of implants are high in low-quality bone, according to a lot of studies [22, 23, 24]. For this reason, it is thought that assessing the bone quality prior to surgery and then having the procedure can further boost the success percentage of the implant. Several writers have attempted to raise the success rate of implants, and have argued that the early stability of the implant is crucial for implant success [25, 26]. An insertion torque between 30 to 60 Ncm is indicative of adequate primary stability, which suggests that the implant will integrate with the bone.It is essential to evaluate bone densities using HU prior to surgery to maximize primary implant stability.

## Conclusion:

The Hounsfield unit helps in identifying bone density of the alveolar ridge and hence it is important to evaluate the HU along with other treatment planning parameters before performing the surgery in order to achieve good primary stability. It is essential to evaluate bone densities using HU prior to surgery to maximize primary implant stability as well.

## Figures and Tables

**Table 1:** : Misch classification of bone density

**Bone classification**	**Description**	**Tactile Sense**	**Bone Density (HU)**	**Localization**
D1	dense cortical bone	oak wood	> 1250	anterior mandible
D2	porous cortical bone and dense trabecular bone	pine wood	850 - 1250	anterior and posterior mandible, anterior maxilla
D3	thin and porous cortical bone and thin trabecular bone	balsa wood	350 - 850	anterior and posterior maxilla, mandible
D4	thin trabecular bone	styroform	150 - 350	posterior maxilla
D5	non mineralized bone	-	<150	-

**Table 2:** Primary stability and houns field unit measured for different implant sites

**Site**	**Number of Implants placed**	**HU (Mean)**	**Torque in Ncm ( Mean)**
anterior maxilla	25	691.6±154.8	35±4
posterior maxilla	25	594±160.2	32±3
anterior mandible	25	820±150.4	45±7
posterior mandible	25	750.56±135	39±6
